# Faecal Microbial Markers and Psychobiological Disorders in Subjects with Morbid Obesity. A Cross-Sectional Study

**DOI:** 10.3390/bs8100089

**Published:** 2018-09-27

**Authors:** Per G Farup, Jørgen Valeur

**Affiliations:** 1 Department of Research, Innlandet Hospital Trust, PB 104, N-2381 Brumunddal, Norway; 2 Unit for Applied Clinical Research, Department of Clinical and Molecular Medicine, Faculty of Medicine and Health Sciences, Norwegian University of Science and Technology, N-7491 Trondheim, Norway; 3 Unger-Vetlesen Institute, Lovisenberg Diaconal Hospital, N-0440 Oslo, Norway; Jorgen.Valeur@lds.no

**Keywords:** gut-brain axis, faecal microbiota, faecal short chain fatty acids, morbid obesity, psychobiological disorders, well-being, mental distress, irritable bowel syndrome

## Abstract

Morbidly obese subjects have a high prevalence of comorbidity and gut microbial dysbiosis, and are thus suitable for the study of gut-brain interactions. The aim was to study the associations between the faecal microbiota’s composition and function and psychobiological comorbidity in subjects with BMI > 40 kg/m^2^ or >35 kg/m^2^ with obesity-related complications. The faecal microbiota was assessed with GA-Map dysbiosis test ™ (Genetic Analysis, Oslo Norway) and reported as dysbiosis (yes/no) and degree of dysbiosis, and the relative abundance of 39 bacteria. The microbiota’s function was assessed by measuring the absolute and relative amount of faecal short chain fatty acids. Associations were made with well-being, mental distress, fatigue, food intolerance, musculoskeletal pain, irritable bowel syndrome, and degree of abdominal complaints. One hundred and two subjects were included. The results confirmed the high prevalence of comorbidity and dysbiosis (62/102; 61%) and showed a high prevalence of significant associations (41/427; 10%) between the microbiota’s composition and function and the psychobiological disorders. The abundant, but in part divergent, associations supported the close gut-brain interaction but revealed no clear-cut and straightforward communication pathways. On the contrary, the study illustrates the complexity of gut-brain interactions.

## 1. Introduction

Obesity is a worldwide health problem that has nearly tripled since 1975 and it affects 13% of the adult population. It is associated with a wide range of comorbidities, such as cardiovascular diseases, diabetes, musculoskeletal disorders, cancer, and psychobiological disorders, and an increased risk of death [1].

Faecal dysbiosis has been defined as an imbalance in the faecal microbiota. It is common in subjects with morbid obesity (MO) and it has been mentioned as a causal factor for obesity and the comorbidities such as insulin resistance, glucose intolerance and diabetes type II, as well as psychiatric and functional disorders [2,3,4,5,6].

The gut-brain axis is a bidirectional link between the gut and the brain and of importance for various psychobiological disorders, such as anxiety, depression, fatigue, stress reactions, pain syndromes, and functional gastrointestinal disorders [7,8,9]. The absolute or relative amounts of the gut microbes per se and the microbes’ metabolic products are possible mediators of the gut-brain effects. Faecal short chain fatty acids (SCFA), which are products of bacterial fermentation, have been proposed as mediators of the health-related effects [10,11].

The primary aim of this study in subjects with morbid obesity was to explore associations between the faecal microbiota’s composition and metabolic products and a selection of psychobiological disorders. The secondary aims were to compare the subjects’ microbiota with that of healthy reference populations. The high prevalence of psychobiological disorders and faecal dysbiosis in subjects with morbid obesity makes this group of special interest for the study of gut-brain interactions.

## 2. Materials and Methods 

### 2.1. Study Design

A cross-sectional study in subjects with morbid obesity. The microbial composition was analysed with a commercially available test, and the results were compared with the test producer’s reference population [12]. The faecal SCFA were compared with the faecal samples from healthy volunteers, as previously published [13].

### 2.2. Participants

Consecutive subjects aged 18–65 years with MO (defined as BMI > 40 or >35 kg/m^2^ with morbidity related comorbidity), referred to the Unit for Obesity at Innlandet Hospital Trust–Gjøvik, Norway in the period from December 2012 to September 2014 were eligible for the study. Subjects with organic gastrointestinal disorders, major psychiatric disorders, severe not obesity-related somatic disorders, alcohol or drug addiction, and previous obesity surgery or other major abdominal surgery were excluded.

The healthy volunteers were healthcare workers and students from Haukeland University Hospital, Bergen, Norway who considered themselves healthy. 

### 2.3. Accomplishment

In all morbidly obese subjects, a medical history was taken, a physical examination was performed, and blood and faecal samples were collected. The information was collected on paper-based questionnaires that were filled in by the doctors, the study nurse, and the participants. Other examinations were performed at the doctors’ discretion. Except for some demographic data, no information was available about the healthy volunteers.

### 2.4. Variables

#### 2.4.1. Participants’ Characteristics

- Gender, age (years), height (m), weight (kg), BMI (kg/m^2^), coffee (cups/day), smoking (daily, previously, never), and previous and present diseases.
- Physical activity was the sum of two questions: Easy activity (not sweaty/breathless): None; <1 h; 1–2 h; >3 h/week (score 0–3). Strenuous activity (sweaty/breathless): none; <1 h; 1–2 h; >3 h/week (score 0, 3, 4, 5). Sum score physical activity 0–8.
- Use of Metformin and other drugs (Yes/No)
- Use of Non-Nutritive Sweeteners (NNS). One unit of NNS was defined as 100 mL NNS-containing beverage or two NNS tablets/teaspoons for use in tea or coffee. A validated food frequency questionnaire that is based on the official Norwegian food composition table was used for the calculation [14].

#### 2.4.2. Psychobiological Disorders

- WHO-5 Well-being index (score 0–100; scores ≤ 50 indicate low mood and scores ≤ 28 indicate likely depression) [15]
- Hopkins symptom checklist 10, (score 1–4; scores ≥ 1.85 indicate mental distress) [16]
- Fatigue (Score 9–63; scores ≥ 36 indicate further evaluation). The diagnose was based on a validated Norwegian translation of the Fatigue Severity Scale [17].
- Musculoskeletal pain from six parts of the body (score 0–12).
- Food intolerance (yes/no) as reported by the participants.
- Irritable Bowel Syndrome (IBS) (yes/no) was diagnosed with a validated Norwegian translation of the Rome III criteria [18].
- Abdominal complaints were scored with IBS Severity Score system (IBS-SSS) (score 0–500) [19]. All of the subjects with abdominal complaints, and not only those with IBS, filled in the questionnaire.

#### 2.4.3. Faecal Microbiota

The CE marked GA-map™ Dysbiosis Test (Genetic Analysis AS, Oslo, Norway) was used for the analyses of the faecal microbiota [12]. The test has a US (Patent No. 9243297) and a European patent (Patent No. 2652145) for its technology governing the oligonucleotide probe set and methods of microbiota profiling [20]. It uses 54 oligonucleotide probes targeting the 16S rRNA gene at different bacterial taxonomic levels.

The overall result is given as the Dysbiosis Index (DI) with scores 1 to 5; values above 2 indicate a microbiota profile that differs from the producer’s reference population (i.e., dysbiosis). The results are also given as the relative abundance compared to a reference population (score −3 to 3) of 39 bacteria at different taxonomic levels (Actinobacteria, Actinomycetales, *Bifidobacterium* spp., Alistipes, *Alistipes onderdonkii*, *Bacteroides fragilis*, *Bacteroides* spp. & *Prevotella* spp., *Bacteroides stercoris*, *Bacteroides zoogleoformans*, *Parabacteroides johnsonii*, *Parabacteroides* spp., Firmicutes, Bacilli, *Catenibacterium mitsuoka*, Clostridi a, *Clostridium* sp., *Dialister invisus*, *Dialister invisus* & *Megasphaera micronuciformis*, *Dorea* spp., *Eubacterium biforme*, *Eubacterium hallii*, *Eubacterium rectale*, *Eubacterium siraeum*, *Faecalibacterium prausnitzii*, Lachnospiraceae, *Lactobacillus ruminis* & *Pediococcus acidilactic*, *Lactobacillus* spp., *Phascolarctobacterium* sp., *Ruminococcus albus* & *R. bromii*, *Ruminococcus gnavus*, *Streptococcus agalactiae & Eubacterium rectale*, *Streptococcus salivarius* ssp. *thermophiles* & *S. sanguinis*, *Streptococcus salivarius* ssp. *Thermophilus*, *Streptococcus* spp., *Veillonella* spp., Proteobacteria, *Shigella* spp. & *Escherichia* spp., *Mycoplasma hominis*, and *Akkermanasia muciniphilia).* The test is a commercial and patented product—hence the dysbiosis scores are the producer’s secret.

In addition, an Alternative Dysbiosis Index (ADI) that is based on the relative abundance of the bacteria Alistipes, Proteobacteria and *Shigella* spp. & *Escherichia* spp., and the relative scarcity of *Bacteroides fragilis*, *Ruminococcus gnavus*, *Bacteroides* spp. & *Prevotella* spp., and *Dialister invisus* was calculated. The ADI has been claimed to separate the favourable dysbiosis (positive scores) from the unfavourable one (negative scores) [21].

#### 2.4.4. Faecal Short Chain Fatty Acids

The subjects with morbid obesity collected the faecal material at home in kits that were provided by the producer of the microbial test and stored it at room temperature for maximum five days before freezing at minus 70 °C [12]. 

Distilled water containing 3 mmol/L of 2-ethylbutyric acid (as internal standard) and 0.5 mmol/L of H_2_SO_4_ was added to 0.5 g of the faecal content and homogenized. According to the method of Zijlstra et al. as modified by Høverstad et al. 2.5 mL of the homogenate was vacuum distilled [22,23]. The distillate was analysed with gas chromatography (Agilent 7890 A; Agilent, CA, USA) using a capillary column (serial no. USE400345H, Agilent J&W GC columns; Agilent, CA, USA) and quantified while using internal standardisation. Flame ionisation detection was employed. The total amount of SCFA and the total and relative amount of acetic, propionic, n-butyric, i-butyric, n-valeric, i-valeric, n-caproic, and i-caproic acids expressed in mmol/kg wet weight and proportion (percentage) were measured and reported. 

The following variables were also calculated:

- Index A (saccharolytic fermentation), which was the concentration of acetic minus propionate minus butyrate divided by the total amount of SCFAs [24].
- Index B (proteolytic fermentation), which was the sum of concentrations of isobutyrate and isovalerate [24].
- The ratio “Propionic acid/Butyric acid”. A high ratio has been proposed as unfavourable [25].

In principle, the analyses of SCFA were performed with identical methods in the subjects with morbid obesity and the healthy volunteers. However, since the analyses were performed in different laboratories and with slightly different preanalytical handling of the samples, only the relative amounts of the SCFA were compared between the groups to avoid bias in the measuring of the total amounts of SCFA.

### 2.5. Statistics

Student *t*-test was used for comparisons between groups, Wilcoxon sign-rank test for comparisons with a reference standard, and linear and logistic regression analyses for the study of associations. In each analysis, all of the cases with data on the relevant variables were included (“available case analysis”). *p*-values < 0.05 were judged as being statistically significant. The analyses were performed with IBM SPSS Statistics for Windows, Version 25.0. Armonk, NY, USA: IBM Corp.

### 2.6. Ethics

The study was approved by the Norwegian Regional Committees for Medical and Health Research Ethics, (reference numbers 2012/966 and 030.08) and was performed in accordance with the Declaration of Helsinki. All the participants gave written informed consent before inclusion. 

## 3. Results

### 3.1. Subject Characteristics

Out of 350 consecutive subjects with morbid obesity, 111 were excluded because the study nurse was unavailable, and 80 refused participation. Out of 159 subjects included in the study, 17 were erroneously included or non-compliant and 40 did not provide faecal samples. Table 1 gives the characteristics of the 102 subjects that were included in this study.

The healthy volunteers that were used for comparisons of the SCFA were four men and eleven women with a mean age of 32.1 years (range 22–68) and BMI 23.7 (range 20.1–27.8 kg/m^2^). 

### 3.2. Dysbiosis Test 

Dysbiosis [Dysbiosis Index (DI) > 2] was present in 62/102 (61%). The mean DI and Alternative Dysbiosis Index (ADI) scores were 2.8 (1.3) and −0.4 (2.6), respectively. When compared with producer’s reference population [12], the relative amount of 22 bacteria were significantly elevated (*p*-values < 0.05, of which 12 bacteria with *p* < 0.001) and the relative amount of 10 bacteria was significantly reduced (*p*-values < 0.05, of which 5 bacteria with *p* < 0.001) The most marked deviations from the reference population were: *Bacteroides* spp. & *Prevotella* spp.: Score 1.59 (1.27) (*p* < 0.001). Bacteroides fragilis: Score 0.54 (0.89) (*p* < 0.001). Bacteroides stercoris: Score 0.44 (0.74) (*p* < 0.001). Eubacterium hallii: Score −0.54 (0.54) (*p* < 0.001). Faecalibacterium prausnitzii: −0.49 (0.71) (*p* < 0.001).

### 3.3. Short Chain Fatty Acids

Total amount of SCFA was 35.99 (SD 21.24) mmol/kg wet weight. Table 2 gives the results in the subjects with morbid obesity and the healthy volunteers with comparisons between the relative amounts of SCFA in the two groups.

### 3.4. Associations between the Psychobiological Disorders and the Microbial Markers

Out of 427 analysed associations between the faecal microbiota and the psychobiological disorders, 41 (10%) were statistically significant. Table 3 gives all of the statistically significant associations between the psychological disorders and the microbiota, and Table 4 provides all of the statistically significant associations between the functional somatic disorders and the microbiota.

## 4. Discussion

The study demonstrated the numerous significant associations between the faecal microbiota’s composition and function and the psychobiological disorders that are challenging to interpret. There is no simple and straightforward explanation and understanding of the gut-brain pathway. The numerous associations indicate complex connections that follow several pathways that are dependent on the trigger and psychobiological disorder.

### 4.1. Associations between the Faecal Microbial Composition and Psychobiological Disorders

A connection between the faecal microbiota’s composition and function and psychological disorders seems to be established, but it is poorly understood [7,9]. The connection is not explained by one or a few species or genus [8]. The multiple associations that are seen in this study indicate a complex regulation of the gut-brain connection. Neither was the DI, a general marker of microbial imbalance, a suitable predictor of all the psychological disorders. The associations between the microbiota and the psychological disorders varied between the disorders. It is unlikely that the three variables that were measured in this study (WHO-5, HSCL-10, and fatigue) are specifically associated with different microbes. HSCL-10 and fatigue were negatively associated with ADI, i.e., associated with an unfavourable dysbiosis. Dysbiosis indices that are based on a combination of microbes might prove to be the best suited predictors of psychological disorders. Similar microbial abnormalities in psychological and functional somatic disorders, indicating common aetiological factors, have been shown in other studies but they were not demonstrable in the current study [26,27].

Some bacteria, such as Faecalibacterium prausnitzii and Proteobacteria, have attracted particular attention [28,29,30,31]. Faecalibacterium prausnitzii was associated with improved well-being (WHO-5) and less mental distress (HSCL-10), supporting the importance of this bacterium. This study did not support previous reports, indicating an association between the phylum Proteobacteria and epithelial dysfunction and risk of disease. Shigella spp. & Escherichia spp. and Proteobacteria were negatively associated with musculoskeletal pain, but not with other psychobiological disorders. 

### 4.2. Associations between the Faecal SCFA and Psychobiological Disorders

Faecal SCFA have been associated with behavioural, psychological and functional somatic disorders and response to treatment [10,11,13,25,32,33]. Like the microbial composition, the results are divergent and in part contradictory and non-reproducible. The favourable effects of butyric acid on brain function were not confirmed in the current study in which total SCFA, acetic acid, propionic acid, and butyric acid were negatively associated with well-being [33]. Previous studies have demonstrated higher levels of faecal SCFA in obese as compared to lean subjects [34,35]. However, the “obesogenic” effect of SCFA remains to be investigated [36].The most noteworthy finding was the associations between IBS and low amounts of total SCFA, acetic acid, iso-butyric acid, iso-valeric acid and valeric acid (both total and relative amount), and reduced proteolytic fermentation. Butyrate has shown favourable effects on visceral sensitivity in healthy volunteers [37]. The local effects of SCFA on the gut seem to be more pronounced than the systemic and centrally mediated ones. The proposed ratio proprionic/butyric acid as a biomarker of IBS was not confirmed [25].

### 4.3. Faecal Microbial Composition and Obesity

As expected, and in accordance with other studies with different methods, the prevalence of faecal dysbiosis measured with the commercially available test was high (61%) [2,3]. Thirty-two out of 39 bacterial groups (82%) deviated significantly from the producer’s reference population. Since diabetes, the use of Metformin and consumption of NNS, which are associated with dysbiosis, were common in the studied population, dysbiosis might have been related to these factors and not to obesity per se [38,39,40,41]. Of note, Faecalibacterium prausnitzii, which has been associated with obesity, was significantly reduced [30].

### 4.4. Faecal SCFA and Obesity

In subjects with morbid obesity, the relative proportions of six out of eight SCFA (75%) deviated significantly from the group of healthy volunteers. In particular, the functions of butyric acid have been studied and seem to be contradictory [42]. In this study, the relative amount of butyric acid was high in the subjects with morbid obesity when compared with the healthy volunteers. In mice, butyric acid reduces appetite and food intake via a central appetite regulation and has a positive influence on the energy balance and diet-induced overweight [43]. If these results are transferable to humans, butyrate has a weight-reducing effect. We are not aware of such studies in humans. Our results did not support this effect. Studies have reported other and opposite effects of butyrate, which in part, could be explained by differences in the metabolic background and dosage [33,42,44].

### 4.5. Strengths and Limitations

The study population was representative of subjects with morbid obesity referred for evaluation of bariatric surgery, and was well suited for this study because of the high prevalence of faecal dysbiosis and psychobiological comorbidity.

Out of the 427 associations between the microbiota and psychobiological disorders (microbiota’s composition: 41 variables; SCFA: 20 variables; and, psychobiological disorders: seven variables), 41 (10%) were statistically significant. The number is higher than expected to occur by chance (type I error). Significant associations do not mean causality, and type II errors are also likely. In such studies, there are numerous unknown confounders, colliders, and mediators, and the analyses were not adjusted for such factors. Therefore, the results of this and similar studies should be interpreted with caution.

The test used for the microbiota’s composition, measuring an undefined dysbiosis index and the relative amount of “only” 39 bacteria at different taxonomic levels might have been inaccurate or incomplete for the purpose of this study. More precise and detailed analytical methods could have given other results.

Since carbohydrates and fibre are major substrates for the microbial SCFA production, the lack of dietary data is another limitation of the study. In subjects with obesity, reduced intake of carbohydrates has been associated with low concentrations of butyrate and butyrate-producing bacteria in faeces [45]. Dietary differences between the subjects with obesity and the healthy volunteers, and not only the differences in BMI, could thus explain the differences in SCFA between the groups. In the gut-brain communication, it is likely that SCFA, which are dependent on the diet, are the mediators of the psychobiological disorders.

The lack of information about the psychobiological disorders in the healthy volunteers, which is a limitation, render analyses of the associations between the faecal markers and psychobiological disorders in this group impossible. The study, therefore, confines itself to a description of the differences in the SCFA profiles between the groups and discusses associations with obesity without mentioning the psychobiological disorders.

Comparisons of the total amount of SCFA, and not only the relative amounts, with the healthy volunteers, could have strengthened the study.

## 5. Conclusions

The current study in subjects with morbid obesity showed a wide range of associations between the faecal microbial markers and psychobiological comorbidity, and thus confirmed the important gut-brain interaction. The study did not clarify simple communication pathways. On the contrary, the study indicated complex and multifactorial relations that often seem contradictory and that need further studies to clarify clinical implications.

## Figures and Tables

**Table 1:** Subject characteristics.

Subject Characteristics	Number (%) Mean and/or Median	SD and/or Range
Gender (male/female)	15 (14.7%)/87 (85.3)	
Age (years)	44.2	8.6
Height (cm)	170	7.8
Weight (kg)	120.8	16.1
BMI (kg/m^2^)	41.8	3.6
Coffee (cups/day)	3.2	2.5
Smoking (daily/previously/never)	14 (13.7%)/46 (45.1%)/42 (41.2%)	
Physical activity (0–8)	4.5	2.3
Diabetes (yes/no)	23 (23.2%)/76 (76.8%)	
Metformin use (yes/no)	16 (18.0%)/73 (82.0%)	
Non-nutritive sweeteners (units *)	7.5 (median 3.3)	10.1 (0–43)
WHO-5 (0–100)	60.4 (median 60)	16 (12–92)
HSCL-10 (1–4)	1.58 (median 1.4)	0.54 (1.0–3.2)
HSCL-10 Mental distress (yes/no)	26 (26.5%)/72 (73.5%)	
Fatigue (6–63)	35.9	14.8
Musculoskeletal pain (0–12)	4.4	2.9
Food intolerance (Yes/No)	55 (55.6%)/44 (44.4%)	
IBS (Yes/No)	27 (27%)/73 (73%)	
IBS Severity scoring system (0–500)	103	0–389

* One unit = 100 mL beverage with non-nutritive sweeteners or 2 tablets/teaspoons for coffee of tea. WHO-5: WHO Well-being index. HSCL-10: Hopkin Symptom Check List 10. IBS: Irritable Bowel Syndrome.

**Table 2:** Short chain fatty acids (SCFA) in subjects with morbid obesity and healthy volunteers. The results are given as mean (SD).

SCFA	Subjects with Morbid Obesity		Healthy Volunteers	MO vs. HV Relative Amounts
	mmol/kg Wet Weight	Relative Amount (%)	Relative Amount (%)	*p*-Value
SCFA total	35.99 (21.24)			
Acetic acid	19.57 (10.72)	55.1 (6.4)	76.9 (9.6)	<0.001
Propionic acid	6.25 (4.16)	17.3 (4.4)	8.5 (3.7)	<0.001
Iso-butyric acid	0.72 (0.61)	2.1 (0.9)	1.4 (0.7)	0.006
Butyric acid	7.13 (5.28)	19.2 (5.3)	9.5 (4.6)	<0.001
Iso-valeric acid	1.05 (0.93)	3.0 (1.5)	2.0 (1.2)	0.017
Valeric acid	0.96 (0.84)	2.6 (1.2)	1.3 (0.8)	<0.001
Iso-capronic acid	0.00 (0.01)	0.0 (0.0)	0.0 (0.0)	0.163
Capronic acid	0.29 (0.51)	0.7 (1.0)	0.4 (0.5)	0.187
Index A	0.19 (0.11)			
Index B	1.77 (1.53)			
Pro/But ratio	1.01 (0.53)	1.01 (0.53)	1.0 (0.4)	0.864

SCFA: Short chain fatty acids. MO: Subjects with morbid obesity. HV: Healthy volunteers. Pro/But ratio: The ratio Propionic acid/Butyric acid.

**Table 3:** The significant associations between the psychological disorders and the faecal microbiota and SCFA. Regression analyses with the psychological variables as dependent variables.

Microbiota	WHO-5		HSC-10		Fatigue	
	B; *p*-Value *	B; *p*-Value Ϯ	B; *p*-Value *	B; *p*-Value Ϯ	B; *p*-Value *	B; *p*-Value Ϯ
Dysbiosis Index	−2.86; 0.024					
ADI			−0.056; 0.011		−1.98; 0.001	−1.81; 0.002
Alistipes					−5.42; 0.022	
*Bacteroides* spp. & *Prevotella* spp.	−3.43; 0.010				2.84; 0.021	
*Bacteroides stercoris*			0.174; 0.019	0.159; 0.028		
Bacilli	4.86; 0.039					
*Dorea* spp.	12.18; 0.014	11.44;0.016				
*Faecalibacterium prausnitzii*	6.37; 0.007	5.65; 0.013	−0.205; 0.011	−0.191; 0.015		
*Phascolarctobacterium* sp.					−6.77; 0.005	−5.94; 0.009
SCFA total	−0.179; 0.019					
Acetic acid	−0.342; 0.024					
Propionic acid	−0.890; 0.022					
Butyric acid	−0.681; 0.026	−0.675; 0.020				

* Linear regression analyses with the psychological variable as dependent variable and one-by-one of the microbiota variables adjusted for gender, age and BMI; Ϯ Stepwise forward linear regression analyses. All the significant variables in the one-by-one analyses were included adjusted for gender, age and BMI.

**Table 4:** The significant associations between the functional somatic disorders and the faecal microbiota and SCFA. Regression analyses with the functional somatic disorders as dependent variables.

Microbiota	Food Intolerance		Musculoskeletal Pain		IBS		IBS-SSS	
	OR; *p*-Value *	OR; *p*-Value Ϯ	B; *p*-Value *	B; *p*-Value Ϯ	OR; *p*-Value *	OR; *p*-Value Ϯ	B; *p*-Value *	B; *p*-Value Ϯ
ADI							−10.86; 0.010	−10.86; 0.010
Actinomycetales			1.34; 0.034					
*Bifidobacterium* spp.			1.22; 0.012	0.94; 0.039				
Alistipes	0.34; 0.019	0.34; 0.019					−40.3; 0.012	
*Alistipes onderdonkii*	0.52; 0.041							
*Bacteroides stercoris*			1.25; 0.001	1.07; 0.004				
*Bacteroides zoogleoformans*					4.64; 0.026	15.55; 0.009		
*Parabacteroides johnsonii*								
*Parabacteroides* spp.					2.10; 0.037	3.31; 0.007		
Firmicutes					2.30; 0.037			
*Dia/ister invisus*					1.95; 0.026	2.91; 0.008		
*Eubacterium rectale*			−1.63; 0.023					
*Phascolarctobacterium* sp.			−1.016; 0.030	−0.85; 0.049				
Proteobacteria			−1.026; 0.050					
*Shigella* spp. & *Escherichia* spp.			−0.71; 0.049	−0.75; 0.030				
SCFA total					0.967; 0.049			
Acetic acid					0.935; 0.033			
Iso-butyric acid					0.080; 0.006			
Iso-valeric acid					0.213; 0.006			
Valeric acid					0.217; 0.012	0.14; 0.003		
Iso-capronic acid			−67.1; 0.034					
Index B					0.379; 0.005			
Valeric acid Pct					0.623; 0.029			
Iso-capronic acid Pct			−27.57; 0.034					
Propionic acid Pct					1.14; 0.021			

* Linear and logistic regression analyses with the functional disorders as dependent variable and one-by-one of the microbiota variables adjusted for gender, age and BMI; Ϯ Stepwise forward linear and logistic regression analyses. All the significant variables in the one-by-one analyses were included adjusted for gender, age and BMI.

## Abbreviations

MO	Morbid Obesity
SCFA	Short Chain Fatty Acids
BMI	Body Mass Index
HV	Healthy Volunteers
DI	Dysbiosis Index
ADI	Alternative Dysbiosis Index
NNS	Non-nutritive sweeteners
OR	Odds Ratio
B	Unstandardized coefficient in the linear regression analyses
WHO-5	WHO Well-being index
HSCL-10	Hopkin Symptom Checklist 10
IBS	Irritable Bowel Syndrome
IBS-SSS	Irritable Bowel Severity Scoring System
